# Supplementary material for: Leaf Lipid Alterations in Response to Heat Stress of Arabidopsis thaliana
Source: Plants (Basel). 2020 Jul 4;9(7):845. doi: 10.3390/plants9070845 (PMC7412450; doi:10.3390/plants9070845)

Figure S1. Selected images of plants in moderate heat stress (main) experiment (below and following). Images pertain to data in Figures 2 through 8 and S3 through S7. These trays contained wild-type plants, *opr3* plants, and several other mutants not described in this paper. The other mutants did not have heat-related phenotypes readily distinguishable from wild-type. Wild-type plants are identified in the legends near the photos. In trays pictured from above, numbering starts in the upper left corner and proceeds across each row.

Path 1 control plants include: **A.** 28-day-old plants at 0 h into heating experiment (left) and at sampling time (right; 16 h at 21ºC; both tray 9). **B.** 28-day-old plants at 0 h into heating experiment (left) and at sampling time (40 h at 21ºC; both tray 15).

Path 2 plants in the experiment incorporating a heat treatment of 12 h at 38ºC and 4 h at 45ºC include: **C.** 28-day-old plants at 0 h into heating experiment (upper left), after 12 h at 38ºC, at which time plants are exhibiting hyponasty (right), and at sampling time (lower left) after 12 h at 38ºC and 4 h at 45ºC (all tray 11). **D.** 27-day-old plants (one day before experiment began, upper left), after 12 h at 38ºC, at which time plants are exhibiting hyponasty (2nd on left and upper right), after 12 h at 38ºC and 4 h at 45ºC (3rd on left), and at sampling time (lower left and lower right) after 12 h at 38ºC, 4 h at 45ºC, and 24 h at 21ºC (all tray 17).

Path 3 plants in the experiment incorporating a heat treatment of 12 h at 21ºC and 4 h at 45ºC include: **E.** 28-day-old plants at 0 h into heating experiment (left) and at sampling time (right and lower left) after 12 h at 21ºC and 4 h at 45ºC (all tray 10). **F.** 28-day-old plants at 0 h into heating experiment (left) and at sampling time (right) after 12 h at 21ºC, 4 h at 45ºC, and 24 h at 21ºC (both tray 16).

A. Path 1 – 0 h (left) and 16 h (right) at 21ºC. They are photos of the same tray (tray 9). Wild-type plants are in positions 8, 14, 24, 51, 53, and 71.

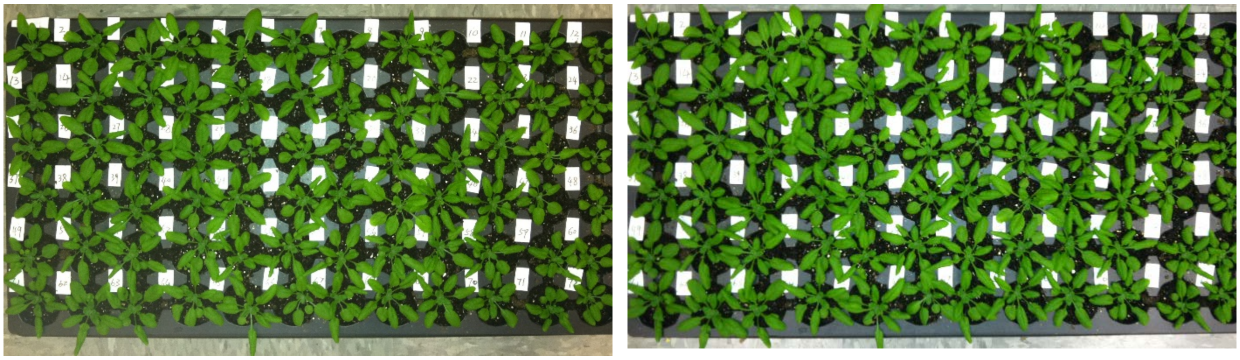

B. Path 1 – 0 h (left) and 40 h (right) at 21ºC. They are photos of the same tray (tray 15). Wild-type plants are in positions 1, 5, 28, 29, 34, and 45.

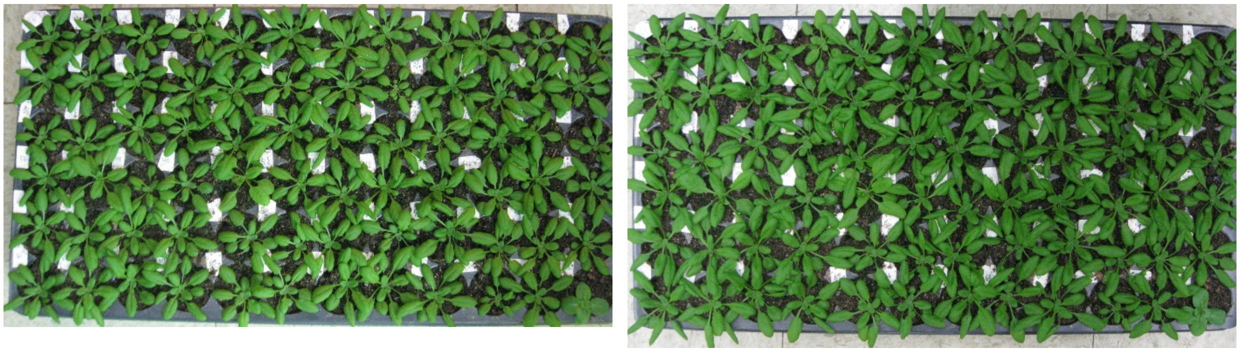

C. Path 2 – 0 h (upper left), after 12 h at 38ºC (right), when plants are exhibiting hyponasty, and at sampling time after 12 h at 38ºC and 4 h at 45ºC (lower left). They are photos of the same tray (tray 11). Wild-type plants are in positions 8, 14, 24, 51, 53, and 71.

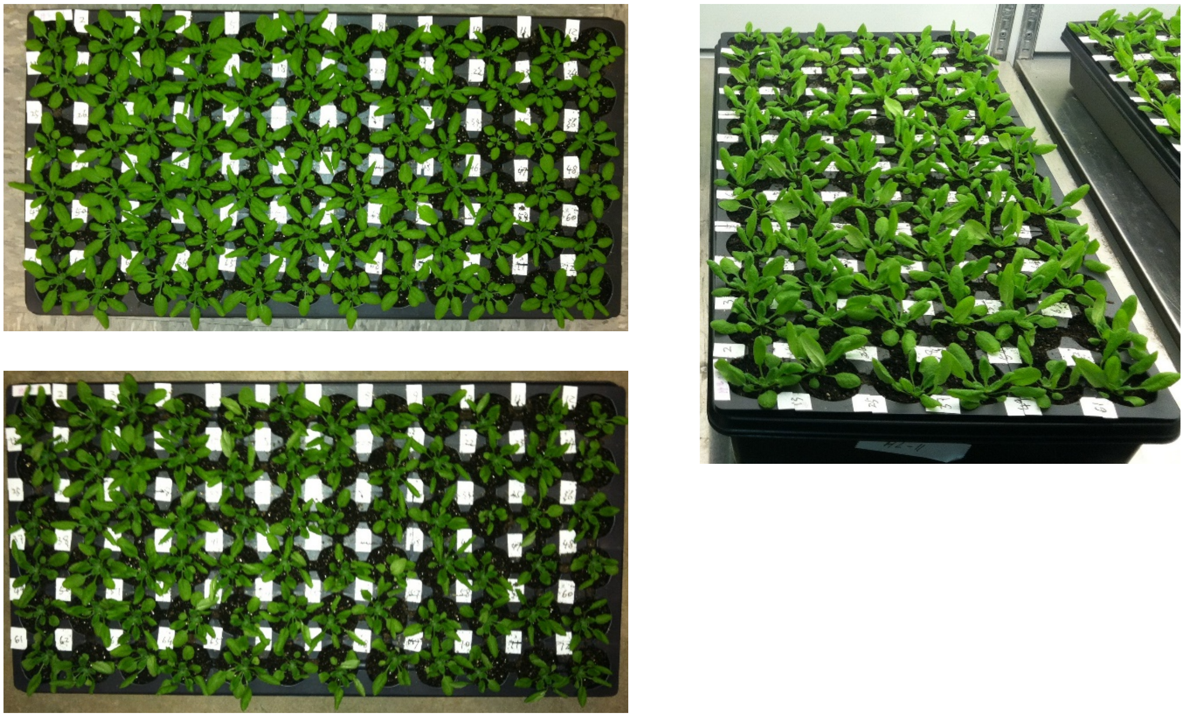

D. Path 2 – One day before the start of the experiment (day 27; upper left), after 12 h at 38ºC (second on left and upper right), when plants are exhibiting hyponasty, after 12 h at 38ºC and 4 h at 45ºC (third on left), and at sampling time after 12 h at 38ºC, 4 h at 45ºC, and 24 h at 21ºC (lower left and lower right). They are photos of the same tray (tray 17). Wild-type plants are in positions 8, 14, 24, 51, 53, and 71.

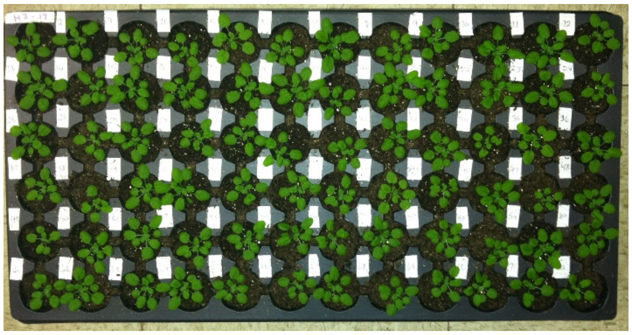

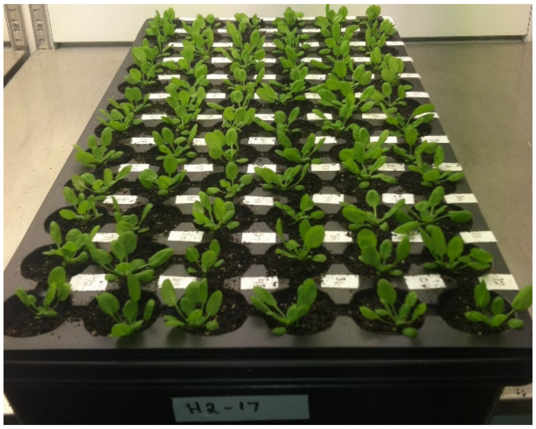

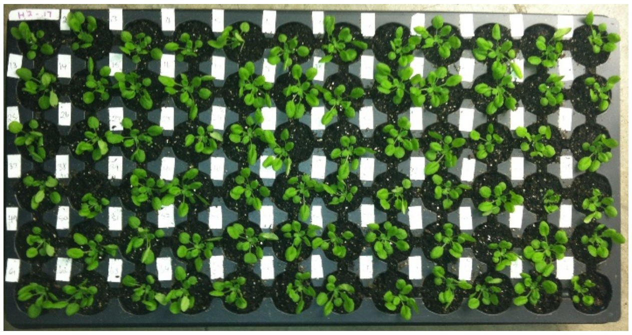

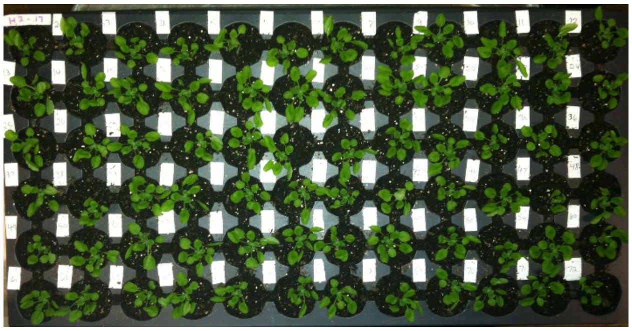

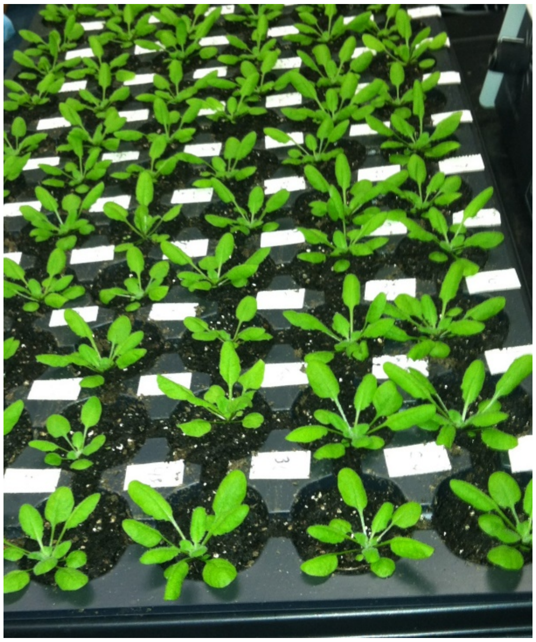

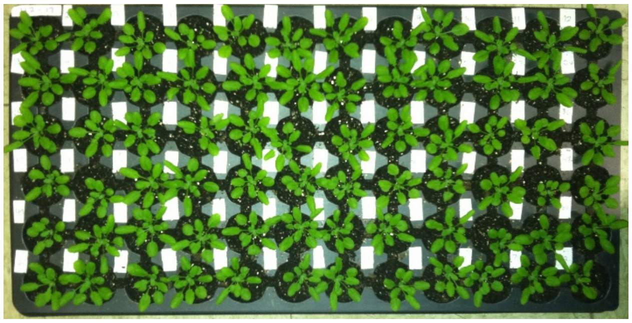

E. Path 3 – 0 h (upper left), and at sampling time after 12 h at 21ºC and 4 h at 45ºC (right and lower left). They are photos of the same tray (tray 10). Wild-type plants are in positions 8, 14, 24, 51, 53, and 71.

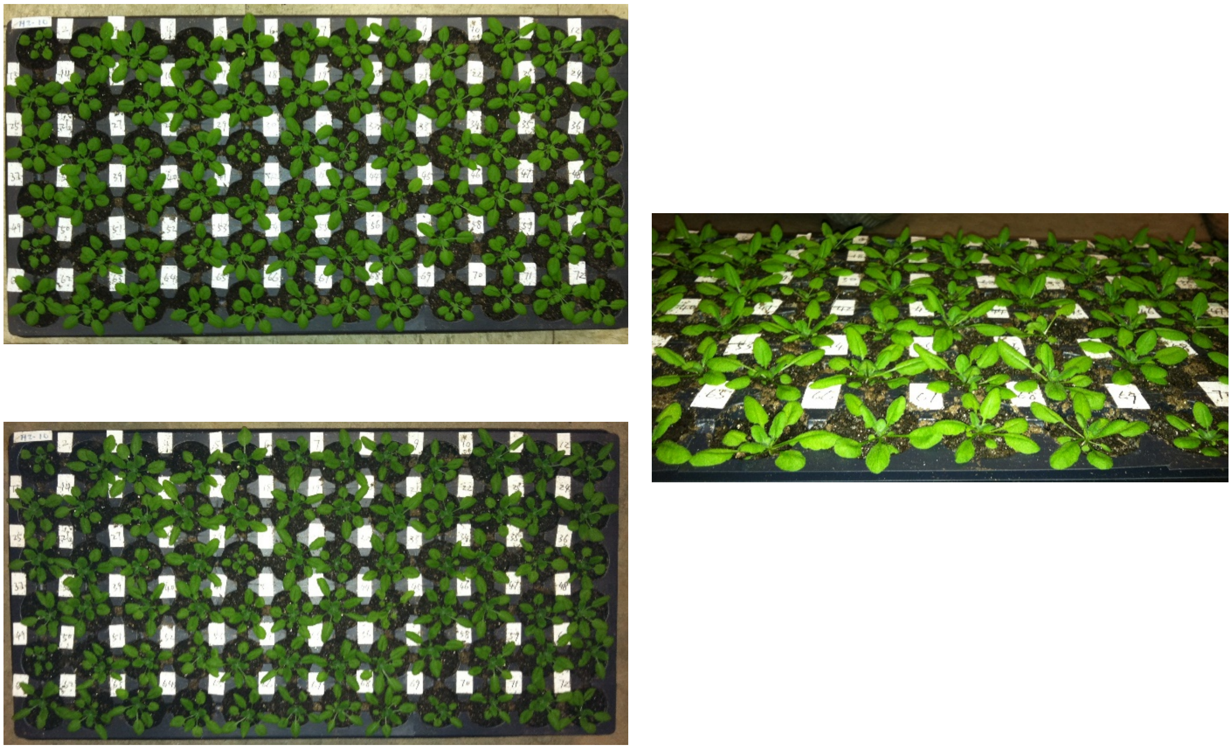

F. Path 3 – 0 h (left), and at sampling time after 12 h at 21ºC, 4 h at 45ºC, and 24 h at 21ºC (right). They are photos of the same tray (tray 16). Wild-type plants are in positions 8, 14, 24, 51, 53, and 71.

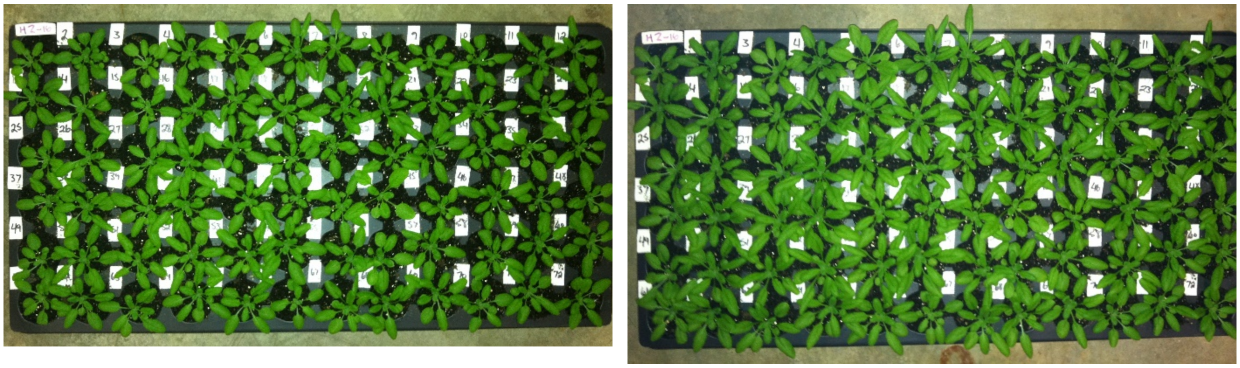

Figure S2. Levels of Arabidopsides in heat-treated *opr3* mutants in comparison to those of heat-treated wild-type plants (below). Lipids from both genotypes were extracted from leaves of plants at the 16-h time point of Path 2, i.e., after treatment of 12 h at 38°C and 4 h at 45°C. Though there is a consistent trend of Arabidopside levels being lower in the mutant, none of the wild-type vs *opr3* comparisons indicate significant differences by Student's t-test. The p-values range from 0.12 to 0.22. Error bars represent standard deviation.

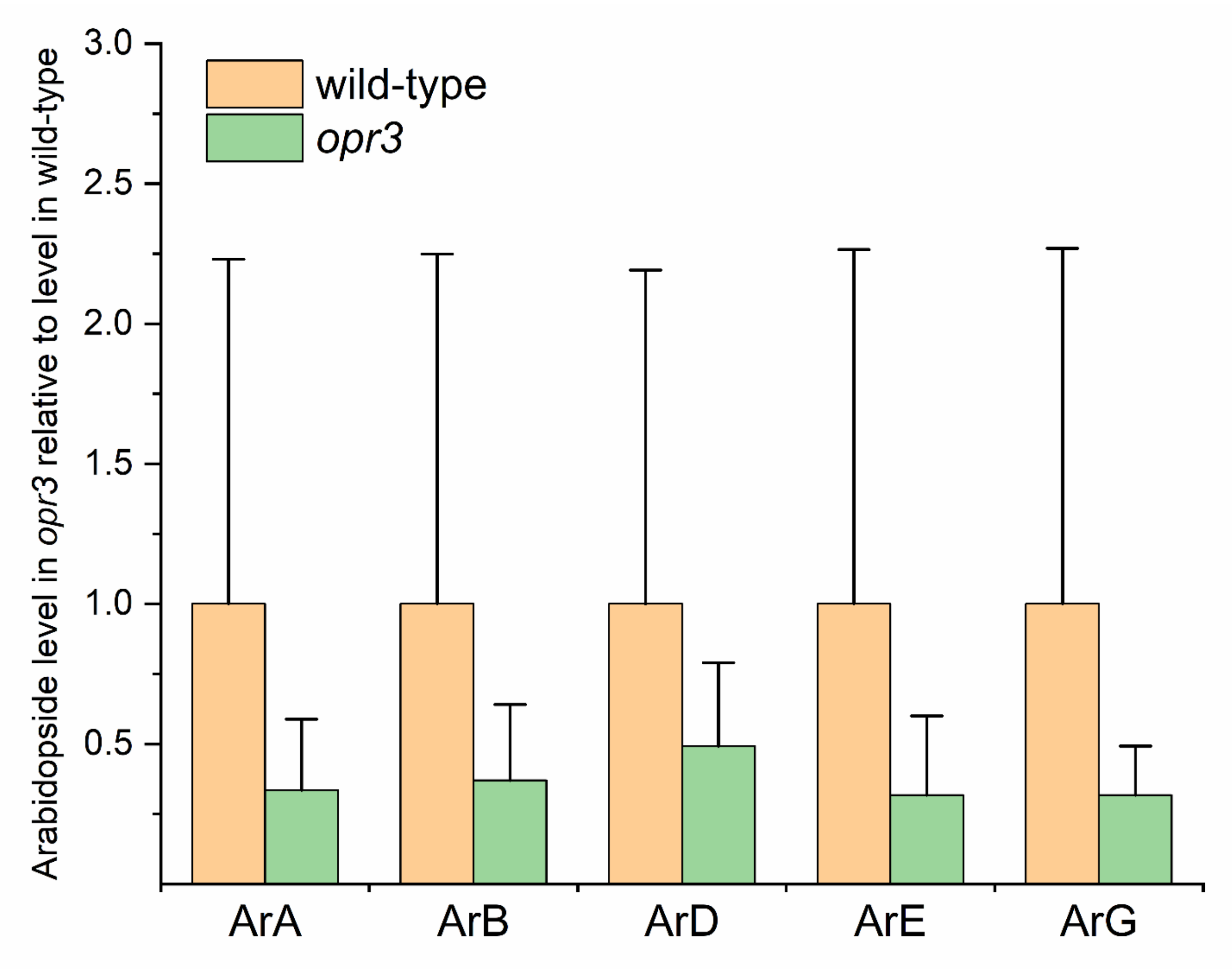

Figure S3. Chain length indices of control and heat-treated diacyl lipid species from leaves of wild-type *Arabidopsis thaliana* (below). Length indices indicate the average number of carbon atoms per acyl chain in each lipid class, calculated as described in Materials and Methods. Comparisons were made at the 16-h time point for control plants (Path 1, orange bars), plants treated for 12 h at 38°C followed by 4 h at 45°C (Path 2, green), and 12 h at 21°C followed by 4 h at 45°C (Path 3, lavender). Asterisks indicate significant differences from that of the Path 1 control plants ($p < 0.05$) by one-way ANOVA, with Tukey's multiple comparisons test, adjusted for FDR. Triangles indicate that a Path 3 value is significantly different than a Path 2 value. Error bars indicate standard deviation.

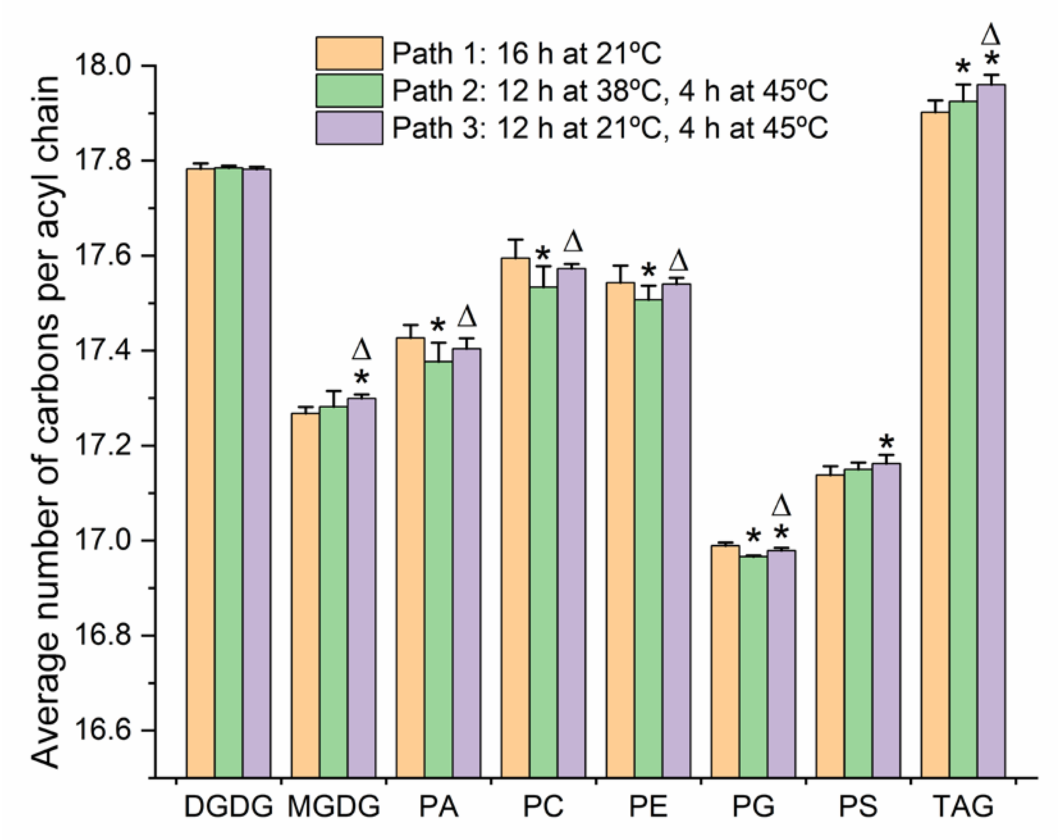

Figure S4. Selected images of plants in the severe heat stress experiment (below). Images illustrate heat stress responses to the treatment used in Figures 9 and 10, Tables 1 and 2, and Tables S8 and S9. Plants shown here were used for the experiments in Tables 1 and 2, i.e., no leaves were removed for lipid analysis. Wild-type plants are in the odd-numbered positions, and *ugtA2,B1* double mutants are in the even-numbered positions for tray positions 1-12, 25-36, and 49-60, while *ugtA2,B1* double mutants are in the odd positions and wild-type plants are in the even positions for positions 13-24, 37-48, and 61-72.

A. Plants at day 30, immediately before heat treatment (top), immediately after treatment at 45ºC for 12 h (middle), and after 12 days of recovery at 21ºC (bottom). These images are all of the same tray.

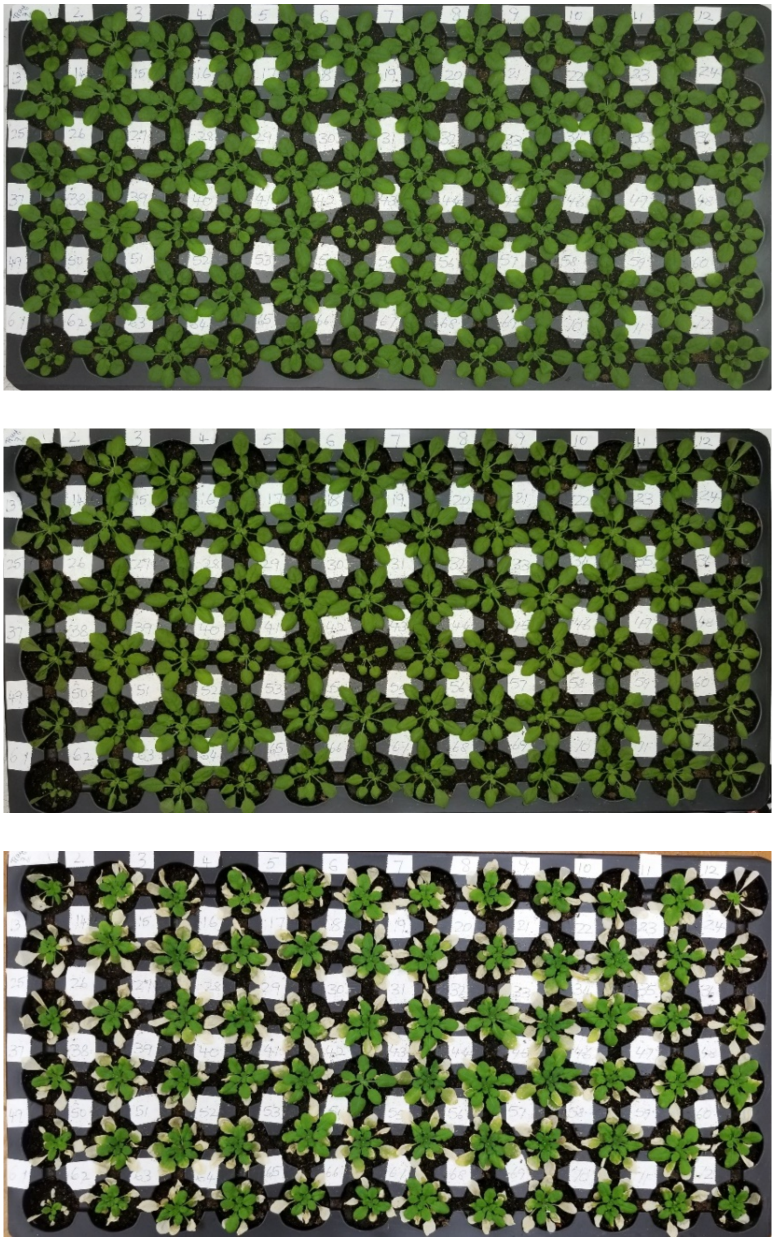

B. Control (untreated) plants at 42 days after growth at 21ºC.

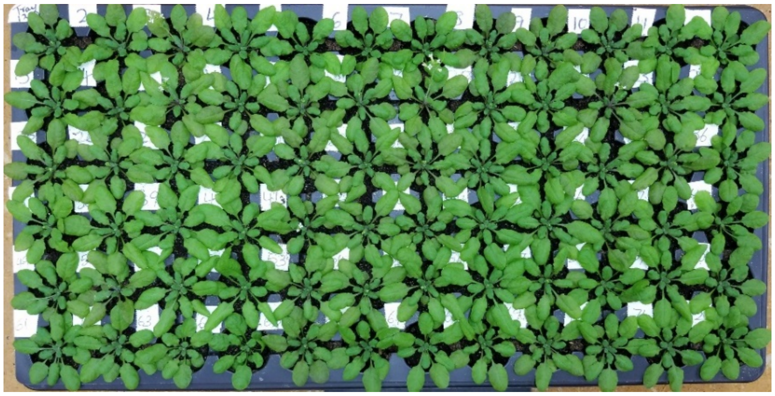

Supplement: Supplementary file 1 [file plants-09-00845-s001.zip › Figures S1-S4.pdf]
